# Relationship Between the Mobility Method and Limb Skeletal Muscle Morphology in the Institutionalized Elderly in Japan: A Study Using Ultrasound Imaging to Evaluate Skeletal Muscle Structure

**DOI:** 10.7759/cureus.98421

**Published:** 2025-12-03

**Authors:** Hideki Ishikura, Kazumi Hiraiwa, Aya Hirao, Junpei Tanabe, Moeko Nakamura, Riho Mende, Ayumi Matsumoto

**Affiliations:** 1 Department of Rehabilitation, Hiroshima Cosmopolitan University, Hiroshima, JPN; 2 Physical Medicine and Rehabilitation, Medical Corporation PIA, Hiroshima, JPN; 3 Physical Medicine and Rehabilitation, Nakamura Hospital, Medical Corporation PIA, Hiroshima, JPN

**Keywords:** activities of daily living, elderly, sarcopenia, skeletal muscle structure, subcutaneous tissue thickness, ultrasound imaging

## Abstract

Background

Reduced mobility among institutionalized elderly individuals is associated with an increased risk of falls, progression to dependence on nursing care, and decline in activities of daily living. However, the qualitative changes in skeletal muscle structure in this population remain poorly understood. This study aimed to noninvasively evaluate muscle morphology, including muscle fiber alignment, as a potential early marker of functional decline.

Methods

This cross-sectional study included 41 elderly residents (mean age, 85.7 ± 8.7 years) of a long-term care facility. Participants were classified into four groups according to their primary means of mobility: independent walking/cane, walker, wheelchair, and stretcher/bed-bound. Physical function was assessed using grip strength, limb circumference, and the Barthel Index. Ultrasound imaging was used to evaluate the muscle thickness, subcutaneous tissue thickness, and myofiber alignment in the upper arm, forearm, thigh, and lower leg. Group comparisons were analyzed using one-way analysis of variance, or Kruskal-Wallis tests with post hoc analysis, as appropriate.

Results

Participants with higher mobility showed significantly greater muscle thickness, grip strength, limb circumference, and Barthel Index scores, along with clear myofiber alignment. In contrast, those with lower mobility exhibited obscured alignment. The lowest-mobility group also demonstrated relatively thicker subcutaneous tissue in the upper arm and forearm.

Conclusions

Maintenance of mobility in institutionalized elderly individuals is associated with both quantitative and qualitative preservation of skeletal muscle. Ultrasound assessment of muscle fiber alignment and subcutaneous tissue thickness may serve as a useful early indicator of sarcopenia, supporting timely preventive interventions.

## Introduction

Reduced mobility in the elderly is associated with increased risk of falls and transition to nursing care and serves as a factor limiting activities of daily living, often progressing to a bedridden status. Previous studies involving the elderly have reported that mobility is associated with survival and walking speed [1]. The progression of sarcopenia associated with decreased physical activity represents a serious problem, particularly among institutionalized elderly individuals, and maintaining mobility is crucial for ensuring independence in life. Sarcopenia is characterized by age-related decreases in muscle mass and strength, and is a typical condition that leads to a decline in functional capacity [2]. Sarcopenia not only has a significant impact on a patient's physical function, but also on hospitalization costs [3], making its prevention and improvement an important issue for society. Individuals with sarcopenia have more than three times the risk of falling [4] and an increased risk of death [5] compared to those without sarcopenia. Decreased muscle mass and strength, which are primarily exacerbated by decreased loading and resistance loads, are particularly pronounced in the muscles of the lower extremities [6]. In institutionalized elderly patients who stand and walk less frequently, muscle mass and strength loss occur in antigravity muscle groups, such as the quadriceps and triceps femoris [7]. Age-related skeletal muscle disorders are further exacerbated by obesity [8], underscoring the importance of managing both skeletal muscle mass and overall body composition. Although previous studies have reported a relationship between muscle mass, muscle strength, and physical function, evaluations of qualitative aspects, such as changes in skeletal muscle structure, have been limited [9]. Few studies have comprehensively examined the extent to which the structural characteristics of skeletal muscles differ among institutionalized older adults with different physical functions, and this remains an important area for future research.

Recent advances in ultrasound imaging technology have enabled the introduction of noninvasive, real-time methods for observing muscle structures in clinical practice, and the utility of these methods has gained considerable attention [10]. Conventional studies have primarily focused on quantitative indices, such as muscle mass and grip strength, as well as quantitative indices such as muscle thickness and cross-sectional area, in muscle assessments using ultrasound imaging [11]. In a clinical setting, observation of the long-axis direction is also practical for intuitive evaluation, as it allows visualization of muscle fiber orientation and alignment. However, knowledge of qualitative aspects, including myofiber arrangement and structural characteristics, remains limited, with no clear criteria or objective scoring; therefore, most evaluations rely on subjective assessments. Accumulating image analysis studies on qualitative changes in skeletal muscles is essential for the clinical application of qualitative muscle characteristics as standardized indices. Previous studies on qualitative muscle characteristics have suggested that muscle weakness is greater than the loss of muscle cross-sectional area, and is associated with increased intermuscular fat [12]. Thus, microstructural changes in skeletal muscles may manifest as an early sign of muscle functional decline and may be useful as an indicator for the early detection of frailty and sarcopenia and for determining the effectiveness of interventions. Although visualizing such microstructural changes using conventional assessment methods proves difficult, recent advances in ultrasound imaging technology have made quantitative assessment methods feasible for muscle thickness and myofiber running [13-15]. Thus, abnormal myofiber alignment may indicate early functional changes; however, its clinical significance and evaluation methods have not yet been established.

This study aimed to identify the early signs of functional decline and provide knowledge for preventive interventions by noninvasively evaluating the structural characteristics of limb muscles using ultrasound imaging and other methods in a group of older adults classified according to their means of transportation within the facility.

## Materials and methods

Study design and participants

This cross-sectional observational study was conducted among the elderly residents of a long-term residential facility. Participants were classified into four groups based on their primary means of transportation within the facility, and the relationships between the structural indices of skeletal muscles, such as muscle fiber arrangement and muscle thickness, and physical function indices, such as grip strength, activities of daily living, and limb circumference, were examined.

**Figure 1:**
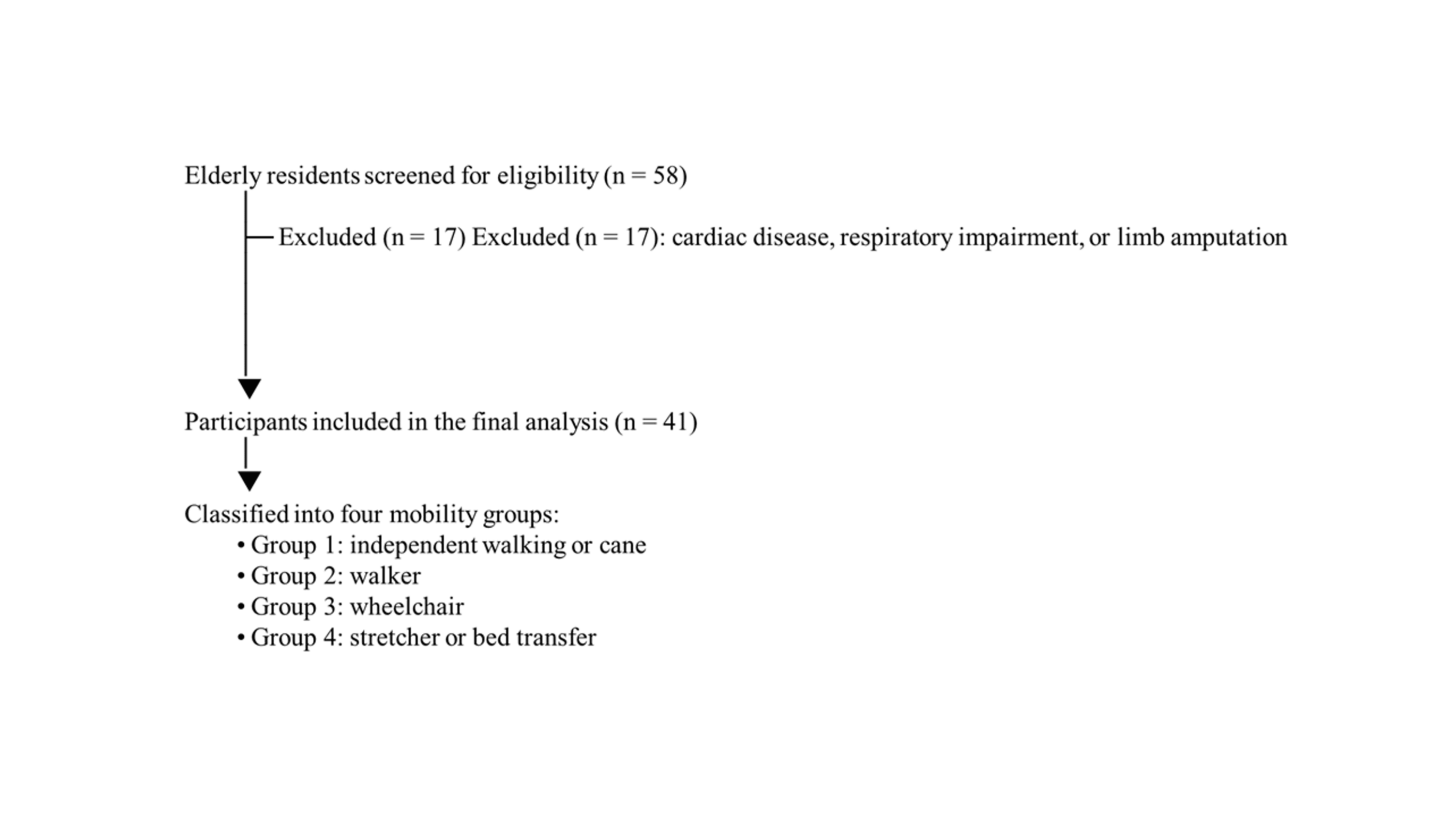
Flowchart of participant screening, exclusion, and inclusion in the final analysis

In this study, 58 elderly patients aged ≥65 years residing in selected long-term residential facilities were screened. Of these, 41 individuals who did not meet the exclusion criteria were included in the final analysis (Figure 1). The exclusion criteria were: (i) not providing consent for the study (n = 0) and (ii) inability to undergo measurements due to cardiac disease, respiratory impairment, or limb amputation (n = 17). Participants were classified into four groups based on their primary means of transportation within the facility: Group 1: independent walking or using a cane; Group 2: using a walker; Group 3: using a wheelchair; and Group 4: transfer by stretcher or while lying in bed.

Assessment of physical function

The following assessment items were evaluated to establish the participants’ physical function and muscle morphology from multiple perspectives.

Assessment of Activities of Daily Living

The Japanese version of the Barthel Index was used to evaluate activities of daily living, derived from the original scale developed by Mahoney and Barthel and published by the Ministry of Health, Labour and Welfare [16-17]. Ten items were evaluated according to participants’ level of independence: eating, transferring, dressing, toileting, bathing, moving, climbing stairs, changing, managing defecation, and managing urination, with a maximum total score of 100.

Grip Strength Measurement

The grip strength of both hands was measured using a digital grip strength meter (Grip-D, Takei Kiki Kogyo Co., Ltd., Aichi, Japan). Maximum grip strength was measured twice while the participants were in a sitting position with both arms hanging toward the body; the maximum value was used as the grip strength value. Grip strength measurement has been widely validated as a reliable indicator of physical function and overall muscle strength in older adults [18].

Limb Circumference Measurements

The circumferences of the right and left extremities were measured to the nearest millimeter using a measuring tape, with the participant in a supine and relaxed position. Limb circumference was measured at the four largest bulges on the upper arms, forearms, thighs, and lower legs in the extended elbow and knee joint positions. Thigh circumference was measured 15 cm above the patella. Limb circumference measurements are commonly used anthropometric indicators with established validity for estimating muscle mass and nutritional status [19].

Muscle Morphology

**Figure 2:**
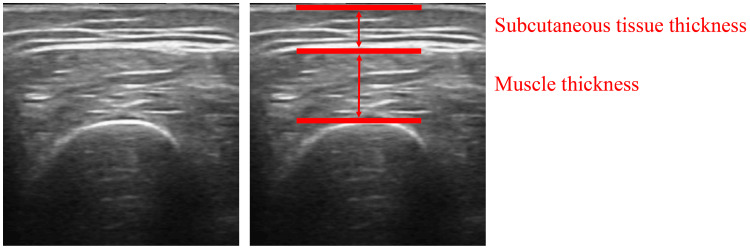
Muscle and subcutaneous tissue thickness measurement

**Figure 3:**
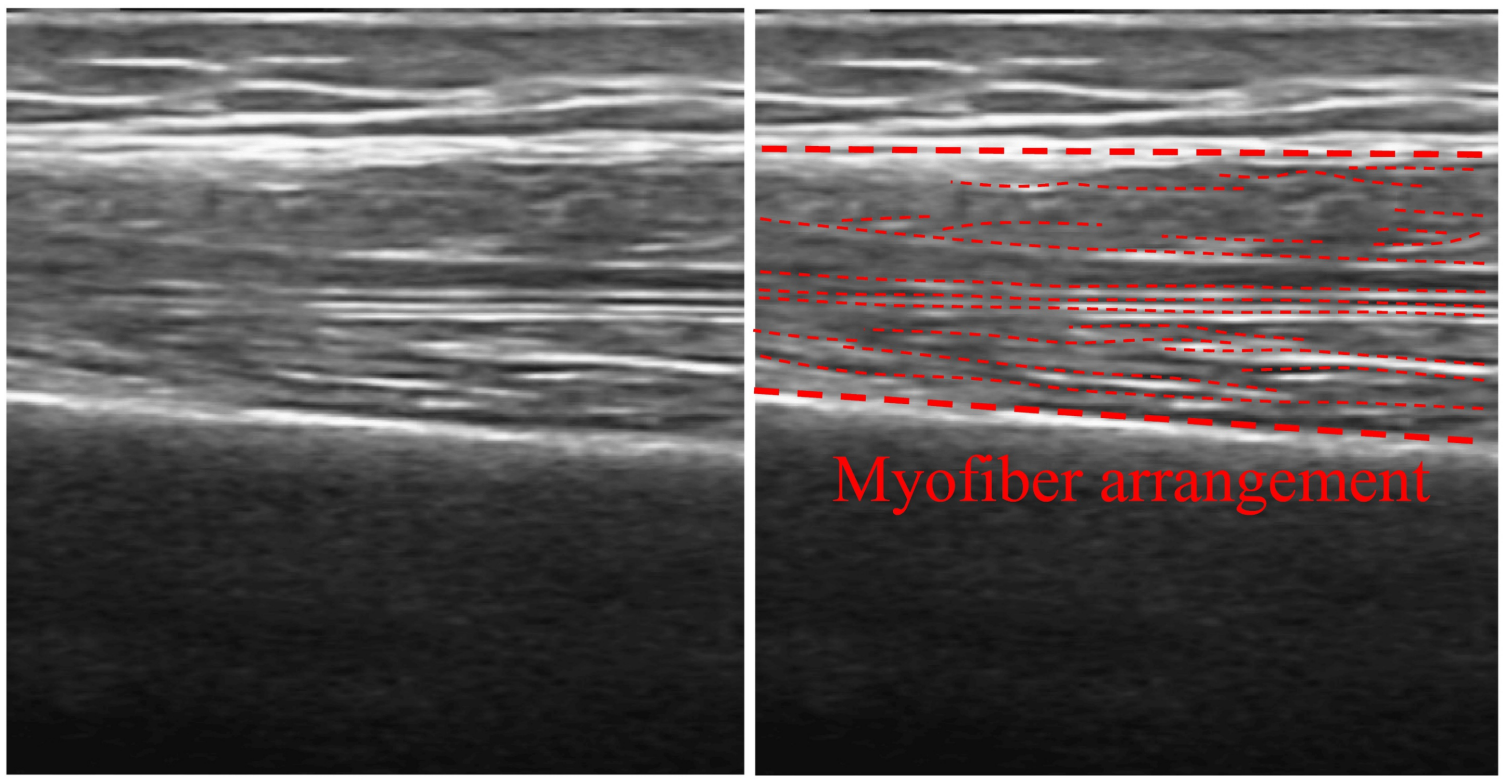
Myofiber arrangement

Muscle thickness and myofiber alignment of the upper arm, forearm, thigh, and lower leg were evaluated using an ultrasound imaging device (FAMUBO-W, Kosei). The ultrasound measurement sites were as follows: center of the anterior surface of the left and right upper arms; maximum bulge of the anterior surface of the forearm; center of the anterior surface of the thigh; and center of the posterior surface of the lower leg. The participants were placed in a supine position with their extremities in natural extension and the probe in perpendicular contact with the skin surface. To measure the lower leg, the lower limb was elevated and extended, a towel was placed under the joint to maintain posture, and a probe contact was made. Muscle thickness was defined as the vertical distance from the bone index to the fascia. Subcutaneous tissue thickness was defined as the vertical distance between the fascia and the skin surface. Each measurement was obtained three times for each site using ImageJ image analysis software, and the average value was calculated (Figure 2). Longitudinal images were acquired for myofiber alignment, and myofiber running was subjectively evaluated (Figure 3). Myofiber alignment was assessed using longitudinal ultrasound images. In each image, the fascicles were visualized as linear structures running between the superficial and deep aponeuroses. The examiner evaluated the alignment subjectively by confirming the continuity and linearity of these fascicle-like echoes across the image. When the fascicle echoes appeared in a parallel and continuous pattern with minimal interruption, the fibers were considered to be properly aligned. All evaluations were performed by the same examiner using consistent image acquisition settings to ensure reproducibility. The movement of the muscle fibers was assessed by the examiner based on the linearity of the movement of the muscle bundles in the longitudinal images. All measurements were performed by the same examiner with careful attention to maintaining consistent probe pressure and positioning. Ultrasound-based assessments of muscle thickness and fascicle alignment have been validated as reliable methods for evaluating skeletal muscle morphology in previous studies [20].

Statistical analysis

A comparative analysis was conducted among the four groups categorized by means of transportation for each endpoint. For each variable, the Shapiro-Wilk test was used to test for normality, and one-way analysis of variance (ANOVA) was applied to variables for which normality was confirmed. Conversely, for variables for which normality was not confirmed, the Kruskal-Wallis test was applied, and when significant differences were found, group comparisons were performed using the Bonferroni method. The significance level for all statistical analyses was set at < 5% (p < 0.05). Statistical analyses were performed using SPSS Statistics version 28.0 (IBM Corp., Armonk, NY, USA). To examine the adequacy of the sample size, we conducted complementary analyses using G*Power (version 3.1.9.7). Post hoc power analyses were computed for each outcome by entering the observed F into the same ANOVA model to obtain the achieved power (1−β).

Ethical statement

This study was conducted in accordance with the Declaration of Helsinki and was approved by the Ethics Review Committee of Hiroshima Cosmopolitan University (approval number: 2019011). All participants or their legal representatives were fully informed of the purpose, procedures, anonymity, and handling of research results. Written informed consent was obtained from all the participants.

## Results

The participants analyzed in this study included 41 older adults (8 men and 33 women) aged ≥65 years residing in a long-term residential facility, with a mean age of 85.7 ± 8.7 years. The participants were classified into four groups based on their primary means of transportation within the facility: Group 1 (independent walking or using a cane, n=8); Group 2 (using a walker, n=6); Group 3 (using a wheelchair, n=14); and Group 4 (stretcher transfer or lying on a bed, n=13). The mean age, height, weight, and Barthel Index of each group are shown in Table 1. Post hoc power analyses indicated that the sample size was adequate for the primary outcomes, with achieved power (1−β) exceeding 0.80.

**Table 1:** Demographics of participants Values are presented as mean ± standard deviation (SD) or number (n). ADL (BI): Activities of Daily Living assessed using the Barthel Index.

	Group 1 (n=8)	Group 2 (n=6)	Group 3 (n=14)	Group 4 (n=13)
Age	83.5 ± 5.8	86.5 ± 9.6	87.8 ± 8.4	84.8 ± 10.3
ADL (BI)	78.8 ± 10.6	79.2 ± 4.9	25.0 ± 20.8	0.4 ± 1.4
Height (cm)	154.5 ± 10.0	145.2 ± 11.9	150.2 ± 11.8	151.2 ± 13.5
Weight (kg)	52.5 ± 5.7	42.1 ± 6.1	44.4 ± 7.7	43.4 ± 10.0

**Figure 4:**
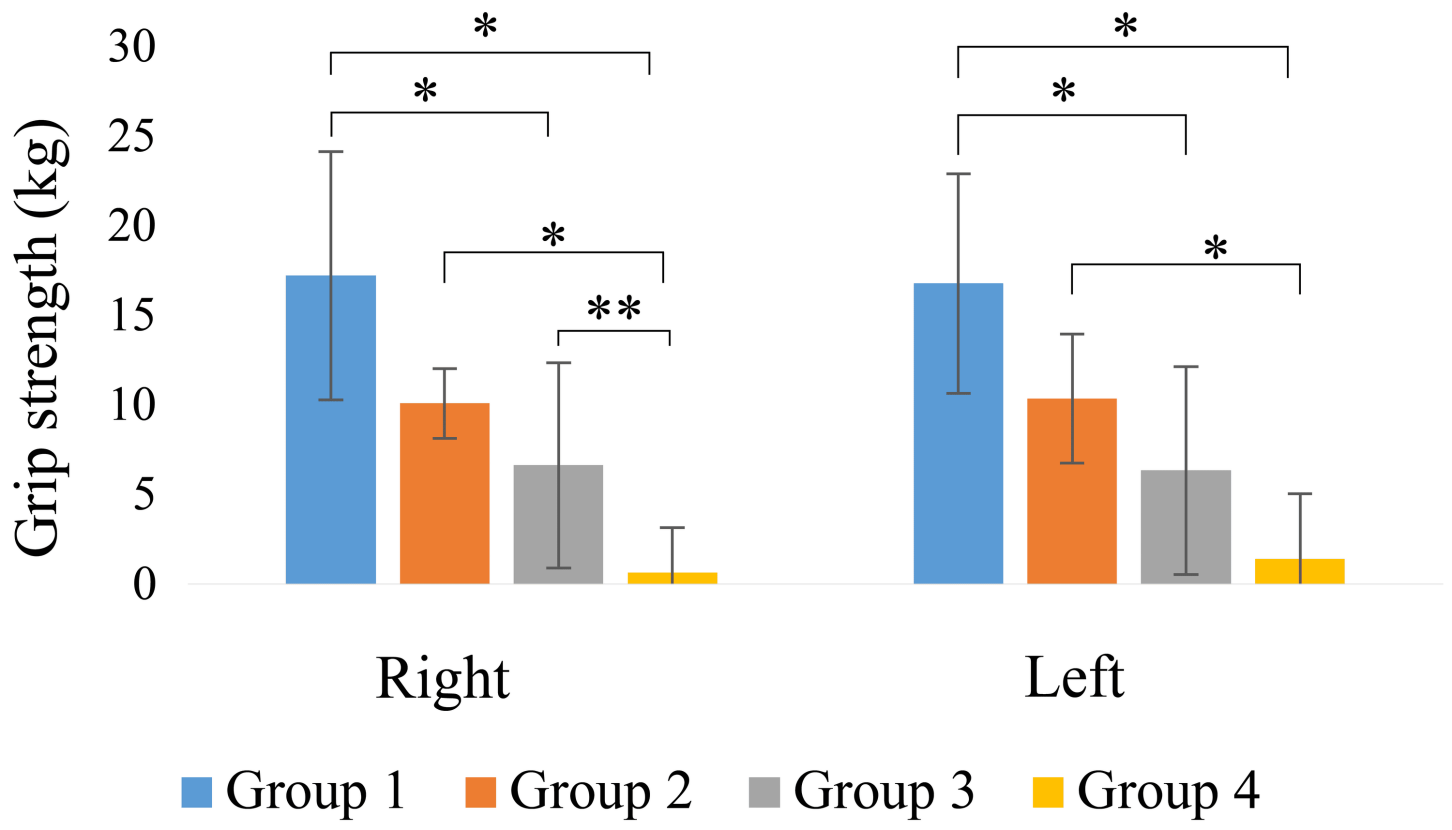
Hand grip strength *: p < 0.01; **: p < 0.05

The left- and right-hand grip strength measurements are shown in Figure 4. Group 1 had a significantly higher grip strength than Groups 3 and 4 (p < 0.05), and Group 2 had a significantly higher grip strength than Group 4 (p < 0.05).

**Figure 5:**
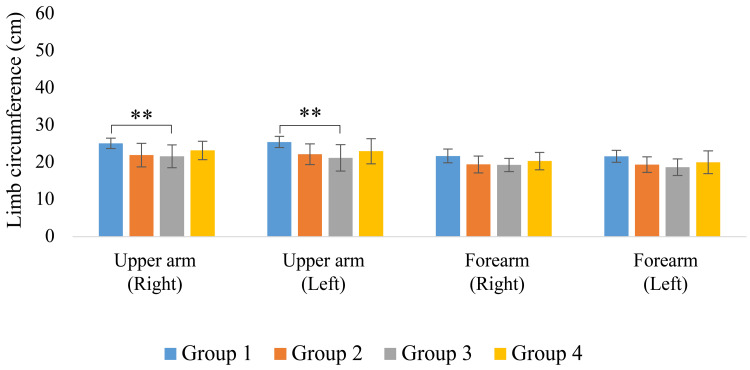
Upper limb circumference ** p < 0.05

**Figure 6:**
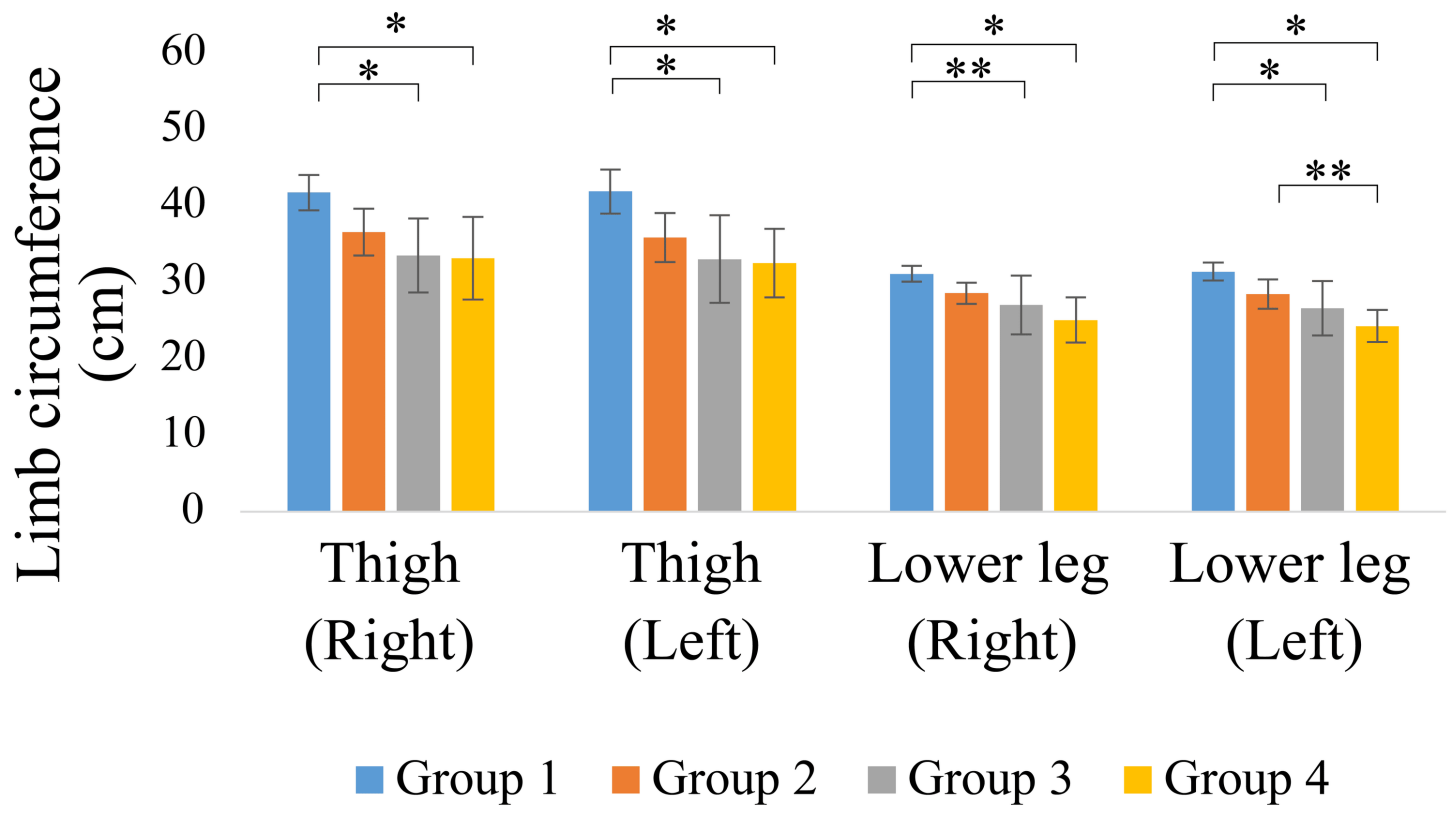
Lower limb circumference *p < 0.01 **p < 0.05

The limb circumference results are shown in Figures 5-6. Groups 1 and 2 showed relatively greater upper arm and thigh circumferences, whereas the lower leg circumference was significantly smaller, especially in Group 4 (p < 0.05).

**Figure 7:**
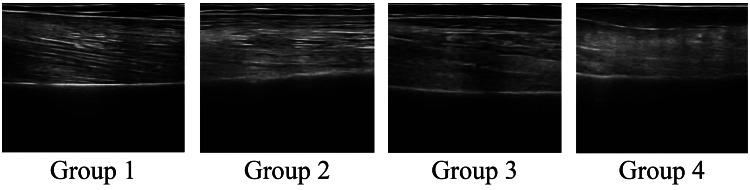
Muscle fiber alignment of the upper arm

**Figure 8:**
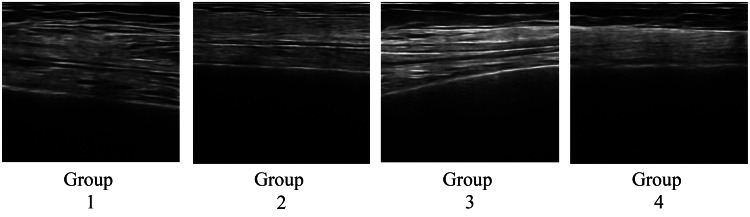
Muscle fiber alignment of the thigh

The myofiber alignment is shown in Figures 7-8. Muscle fibers in the upper arm and thigh ran relatively parallel in Groups 1 and 2, whereas in Group 4, the fiber alignment was unclear, and visibility was significantly reduced.

**Figure 9:**
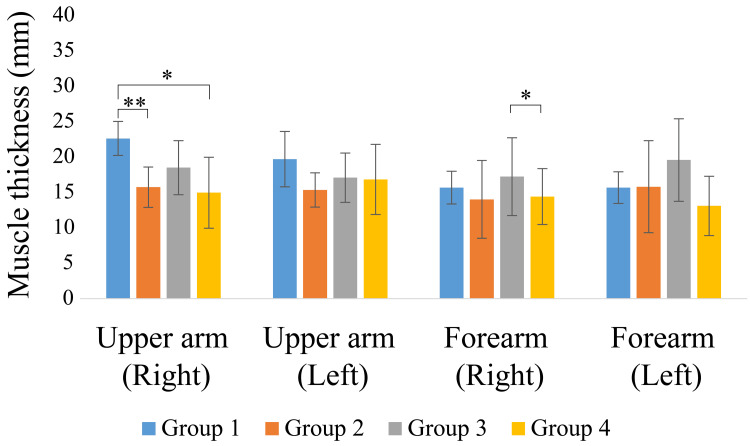
Upper limb muscle thickness * p < 0.01; ** p < 0.05

**Figure 10:**
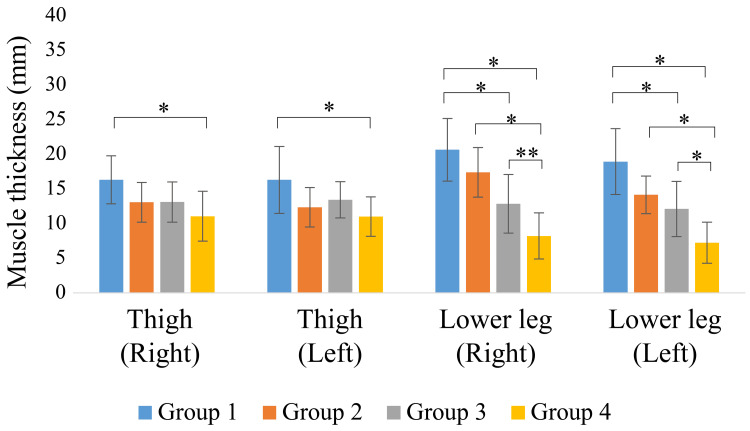
Lower limb muscle thickness * p < 0.01; ** p < 0.05

**Figure 11:**
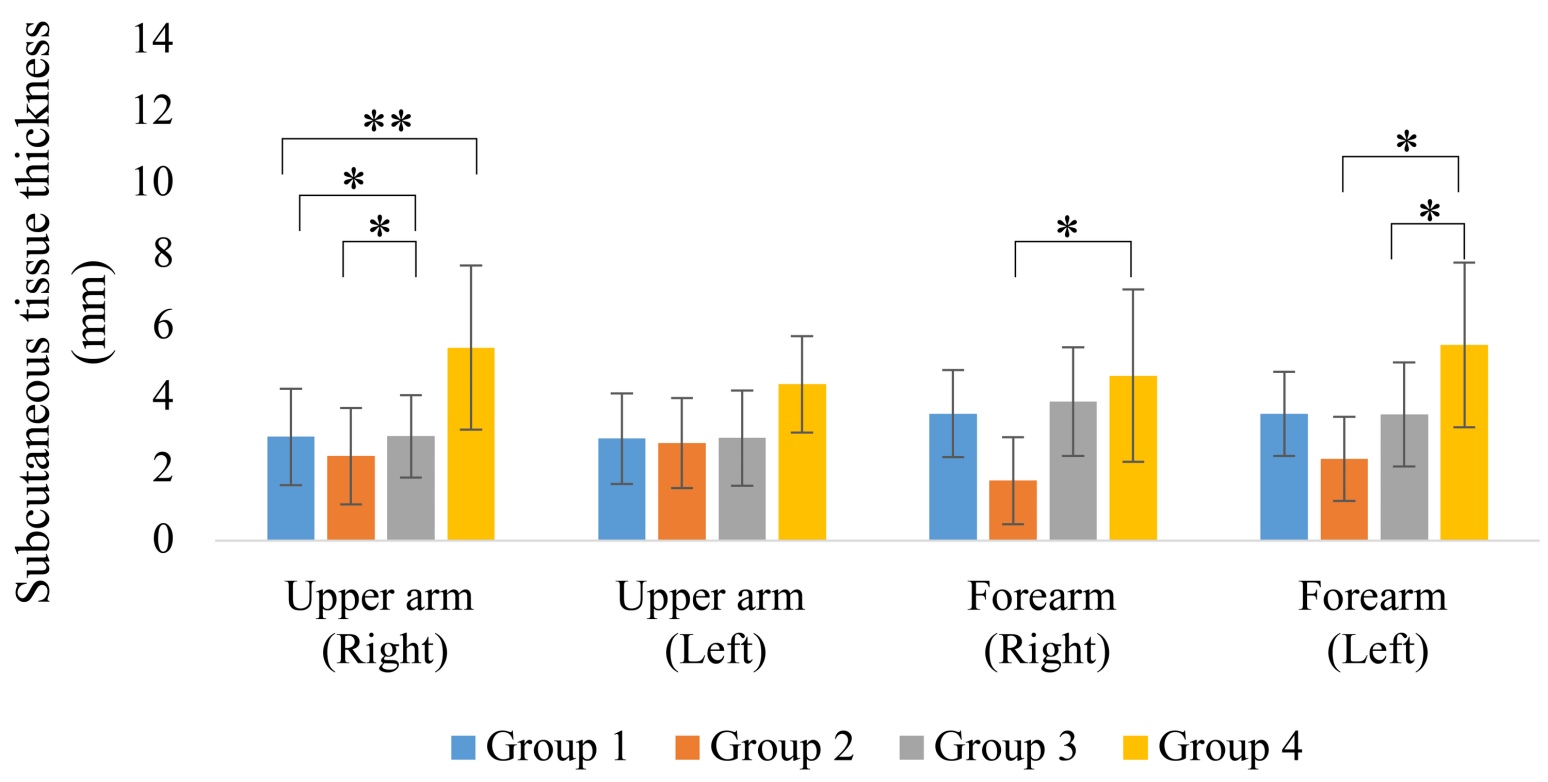
Upper limb subcutaneous tissue thickness * p < 0.01; ** p < 0.05

**Figure 12:**
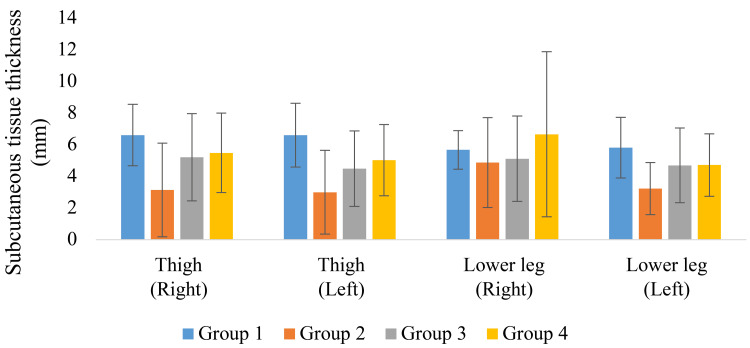
Lower limb subcutaneous tissue thickness

The measurements of muscle and subcutaneous tissue thickness are shown in Figures 9-12. Muscle thickness of the thighs and lower legs was higher in Group 1 than in the other groups, and a significant difference was observed between Groups 1 and 4 (p < 0.05). Lower leg muscle thickness was significantly greater in Group 1, followed by Group 3 and Group 4. Meanwhile, the muscle thickness of Group 2 was significantly greater than that of Group 4 (p < 0.05). The subcutaneous tissue thickness of the upper arm and forearm tended to be greater in Group 4 than in the other groups.

## Discussion

In this study, institutionalized elderly residents were classified based on their primary means of mobility within the facility and quantitative and qualitative indices of physical function and skeletal muscle structure were compared. The results showed that the highly mobile groups (Groups 1 and 2) had significantly higher values for muscle thickness, grip strength, limb circumference, and Barthel Index, as well as a clear arrangement of muscle fibers. In contrast, the groups with lower mobility (Groups 3 and 4) generally had lower values for these indices, and the arrangement of muscle fibers was unclear. These results suggest that differences in mobility may influence the structural maintenance of skeletal muscles and the preservation of physical function in institutionalized elderly residents.

In the longitudinal ultrasound images, myofibers appeared as hyperechoic linear structures running between the superficial and deep fascia. Groups 1 and 2 showed relatively parallel and continuous fascicle-like echoes, whereas Group 4 exhibited fragmented, poorly defined, or discontinuous fascicle patterns, indicating reduced clarity of architectural organization. These qualitative findings complement the quantitative measurements of muscle thickness and suggest that reduced mobility may negatively influence both structural clarity and muscle architecture. In terms of qualitative changes in skeletal muscle, more clearly defined myofiber runs were observed in the highly mobile group, whereas obscured sequences were noted in the less mobile group in the lower extremity muscles (thighs and lower legs). This may reflect the effects of loading stimuli, such as standing and walking, on muscle structure. Fukumoto et al. [21] reported that increased physical activity mitigated future decline in thigh muscle thickness, and Lopez et al. [22]found an association between echo intensity and standing ability on the ultrasound images of the quadriceps muscle. These findings indicate that differences in mobility may influence quantitative and qualitative differences in lower extremity muscle structures. A unique feature of this study is the inclusion of the qualitative aspect of myofiber arrangement in addition to the measurement of muscle thickness as an evaluation target. Traditionally, the evaluation of muscle structure tends to be biased toward muscle mass (muscle thickness and cross-sectional area); however, recent studies have indicated that muscle weakness cannot be explained simply by a decrease in muscle mass alone and that “qualitative changes,” such as intermuscular fat and fiber arrangement, are also involved [12]. The obscured myofiber alignment observed in the low-mobility group may reflect a disruption in the structural order of the muscle, which was particularly evident in the group with less frequent muscle contractions, and may be an effect of the lack of mechanical stimulation on the intramuscular structure.

Regarding body composition other than skeletal muscle, subcutaneous tissue thickness was also evaluated in this study, and a trend toward increased subcutaneous tissue thickness in the upper arm and forearm was observed, especially in Group 4. The elderly in the facility often performed many activities of daily living with assistance, especially those in Group 4, whose activity was extremely low and voluntary use of the upper limbs was greatly restricted. Such a state of nonuse may induce muscle atrophy and hypometabolism and manifest in the form of local accumulation of adipose tissue. The simultaneous loss of muscle mass and accumulation of fat is suggested to be an accelerating factor in the functional decline in sarcopenic obesity [23]. The increase in subcutaneous tissue thickness in Group 4 participants in the present study can also be interpreted as a form of pathological restructuring due to reduced muscle use rather than mere overnutrition. In addition, a study by Hwang et al. reported that the frequency of muscle contractile activity may affect local lipolysis [24]. Thus, the accumulation of subcutaneous fat, especially in the upper extremities as observed in the present study, may be closely related to a bias in activity patterns.

Conventional indices, such as grip strength and limb circumference, also tended to be significantly better in the group with higher mobility. Grip strength is also an indicator of the overall muscle strength and is highly effective in detecting age-related sarcopenia at an early stage [25,26]. In addition, a decrease in limb circumference is strongly associated with a decrease in muscle mass, and leg circumference is considered a sensitive indicator of a non-weight-bearing status. The results of this study have high clinical significance.

Overall, the results of the present study suggest that mobility measures performed in a facility are closely related to the maintenance of normal skeletal muscle structure and physical function. Among others, muscle fiber alignment observed by ultrasound is expected to be clinically applicable as a noninvasive method for visualizing qualitative changes in muscles, suggesting its usefulness in future rehabilitation assessments. With regard to structural changes in skeletal muscle, early detection would contribute to optimizing the timing of interventions related to the prevention and improvement of sarcopenia, as well as to individualized rehabilitation design based on a person's movement performance status.

However, this study has some limitations. First, the cross-sectional observational design of the study precluded establishing a causal relationship between muscle structure and physical function. Therefore, investigating whether structural changes in skeletal muscles are associated with changes in movement execution is necessary. Second, the assessment of muscle fiber alignment involves subjective judgment, and eliminating the possibility of rater bias proves difficult. More objective scoring is required to evaluate muscle fiber sequences in clinical situations. Third, because the participants comprised a relatively limited population from a single institution, generalization of the results requires careful interpretation. Surveys involving a wide range of participants, including those from multiple facilities with balanced numbers of men and women, are required. Establishing an objective scoring system for muscle fiber array evaluation and the accumulation of large-scale data through multicenter collaborative studies is warranted. In addition, developing quantitative and reproducible indices for qualitative evaluation through automated ultrasound image analysis and AI-assisted methods may enhance the accuracy of early frailty and sarcopenia detection and improve intervention strategies.

## Conclusions

This study examined the relationship between physical function and skeletal muscle structure in elderly residents of a long-term care facility in relation to their daily mobility. Participants with high mobility exhibited more favorable qualitative characteristics, such as clearer muscle fiber alignment and thinner subcutaneous tissue, alongside superior quantitative indices, including greater muscle thickness, grip strength, limb circumference, and Barthel Index scores. Conversely, those with lower mobility demonstrated overall functional decline and degenerative changes in muscle structure. These findings highlight the importance of maintaining mobility to preserve both the quantitative and qualitative aspects of skeletal muscle. Evaluating structural indices, such as myofiber arrangement and subcutaneous tissue thickness, may serve as a useful noninvasive approach for the early detection of sarcopenia and the prediction of functional prognosis. Future studies should focus on developing and standardizing quantitative ultrasound-based assessment methods and establishing qualitative evaluation models to support early intervention and risk management for physical function decline in the elderly.
